# The factors associated to psychosocial stress among general practitioners in Lithuania. Cross-sectional study

**DOI:** 10.1186/1472-6963-5-45

**Published:** 2005-06-10

**Authors:** Giedrius Vanagas, Susanna Bihari-Axelsson

**Affiliations:** 1 Preventive Medicine dept., Kaunas University of Medicine, Kaunas, Eiveniu 4, Lithuania; 2 Nordic School of Public Health, Gothenburg, Nya Varvet 25, Sweden

## Abstract

**Background:**

There are number of studies showing that general practice is one of the most stressful workplace among health care workers. Since Baltic States regained independence in 1990, the reform of the health care system took place in which new role and more responsibilities were allocated to general practitioners' in Lithuania. This study aimed to explore the psychosocial stress level among Lithuanian general practitioner's and examine the relationship between psychosocial stress and work characteristics.

**Methods:**

The cross-sectional study of 300 Lithuanian General practitioners. Psychosocial stress was investigated with a questionnaire based on the Reeder scale. Job demands were investigated with the R. Karasek scale. The analysis included descriptive statistics; interrelationship analysis between characteristics and multivariate logistic regression to estimate odds ratios for each of the independent variables in the model.

**Results:**

Response rate 66% (N = 197). Our study highlighted highest prevalence of psychosocial stress among widowed, single and female general practitioners. Lowest prevalence of psychosocial stress was among males and older age general practitioners. Psychosocial stress occurs when job demands are high and job decision latitude is low (χ^2 ^= 18,9; p < 0,01). The multivariate analysis shows that high job demands (OR 4,128; CI 2,102–8,104; p < 0,001), patient load more than 18 patients per day (OR 5,863; CI 1,549–22,188; p < 0,01) and young age of GP's (OR 6,874; CI 1,292–36,582; p < 0,05) can be assigned as significant predictors for psychosocial stress.

**Conclusion:**

One half of respondents suffering from work related psychosocial stress. High psychological workload demands combined with low decision latitude has the greatest impact to stress caseness among GP's. High job demands, high patient load and young age of GP's can be assigned as significant predictors of psychosocial stress among GP's.

## Background

Lithuania is one of the three Baltic States which regained independence in 1990. Back in 1989 a Congress of Physicians of Lithuania took place in which the necessity to reform the health care system was discussed. To implement these reforms a National Health Care Conception was adopted in 1991 by the Parliament. The main goal of the reform is to optimise the health care resources and services for better health of the population. The development and reformation of Primary Health Care was underlined as a key factor of a whole Health Care Reform. The main concept argues development of the primary health care services reorienting them from disease centered episodic activities to patient needs, continuity, comprehensiveness, health promotion and disease prevention.

Primary health care services in Lithuania are delivered in primary health care centers, GP's, both school and community medical posts (paramedical centers), ambulatories and polyclinics, women's consultancies, nursing hospitals, as well as by the ambulance service (stations and divisions). A health reform goal was that all practicing physicians in primary health care (PHC) level (district internists and pediatricians) should be replaced by family doctors (GPs) by 2005.

More than 300 public and private GP clinics are in operation. At the moment, the vast majority of health care facilities are publicly owned, but there are plans to partially privatize primary care. For the most part private primary care takes the form of solo or small group physician-owned practices [1]. Currently, more than 1000 GPs practicing in primary health care. Many GP's have more than 2000 patients in the list when the reform statement it is suggested that an appropriate average should be of 1600 patients per one GP. Not all of GP graduates are practicing family medicine; there is still lack of GP's in rural areas. A financing principle of the health care system is based on compulsory health insurance and does not support to cover practice needs. PHC services are covered by capitation fees only, which average is about €20 per capita per year.

In 1996–1997 operational service standards for GP's were defined. To GP's services were defined new task to deliver pediatry, gynaecology and many other services as primary health care. Since 1998, an existing partial gate-keeping role for family doctors was switched in 2002 to complete gate keeping. This new role tasks increased workload and responsibilities for Lithuanian GP's. The primary role of the Lithuanian GP today continues to be in diagnosis and ongoing management of medical conditions, with consultations accounting for about 50% of their workload. GP's are overloaded by patient's list and paperwork, consuming other 50% of their working time.

Number of studies has been argued that general practice has become an increasingly stressful place to work [2-11] by the increasing demands and constraints [6,9,12-17]. It is showed that about half of the investigated general practitioners (GP's) were not satisfied with their work [18-22] due to high job requirements. In recent literature important sources of psychosocial stress for GP's are mentioned: excessive paperwork, health reforms, bureaucratic interference (6), job demands, decision latitude [9], workplace location [23] job pressure, patient load [6,18,24,25], lack of organizational support [26-30], dealing with difficult patients [31,32] and objective personal characteristics such as age, gender and workers marital status [33-36].

Independence and flexibility as provided by the continuing Lithuanian health care reform with regard to the primary care services as a small business are now being undermined by high workload requirements to general practitioners [9,37] due to new tasks, excessive paperwork and high patient load, which can lead to intension to quit general practice, lower intensions to attend higher professional standards, and can take turnover to quality of care and to patient's satisfaction with services [5,6,32,38-40].

Aim of this paper is to explore the psychosocial stress level among Lithuanian general practitioners and to examine the relationship between psychosocial stress and work characteristics. Main investigated research questions were: What is prevalence of psychosocial stress regarding the sociodemographic characteristics of GP's? How do work demands and decision latitude influence the presence of psychosocial stress and low quality of life among GP's? What characteristics can be predictors of psychosocial stress among GP's?

## Methods

### Target group

Lithuanian general practitioners.

### Study design

Cross – sectional study. A mailed survey of random national samples. Computerized randomization was performed from the registry of Lithuanian physicians. The data collected through the questionnaires filled in by the GP's.

### Questionnaire

The study involved the development and administration of a questionnaire for GPs. The questionnaire was designed using the Karasek and Reeder. A questionnaire was pilot tested among a group of ten GPs.

### Sample size

Sample size was calculated using EpiInfo 2000 Statcalc software whish argued the sample size of 192 GP's with the 95% confidence level. From the previous studies the expected response rate was 63%. Therefore, it was decided to send questionnaires to 300 Lithuanian GP's. Our observed response rate was 66%. We collected 197 filled in questionnaires.

### Assessmen of psychosocial stress

Psychosocial stress in this study was investigated by a questionnaire based on the Reeder scale [41,42]. The Reeder scale uses four statements experienced in everyday stressful situations as "usually tense or nervous", "daily activities are extremely trying and stressful". The respondents should indicate whether each of the statements describe them. Each question has four alternative responses, which were coded using Likert-like scale. For scoring we used the simple summation method [43].

### Assessment of work characteristics

**Figure 1:**
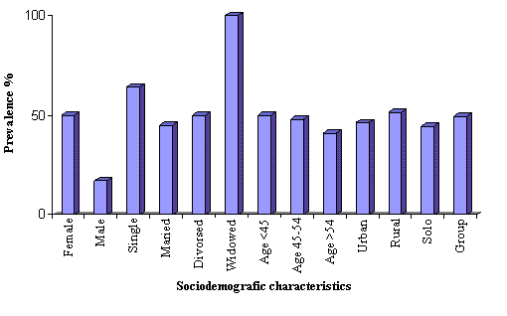
Prevalence of psychosocial stress among GP's by sociodemographic characteristics.

Work characteristics were investigated with the Karasek scale [44,45]. This model, also known as the "job strain" model. (fig. 1)

The Lithuanian version of Karasek's scale of 11 questions was adopted by prof. A. Gostautas in 1992. This scale measures job character – decision latitude and psychological workload demands.

The first scale – decisions latitude scale is composed of two subscales: skill discretion and decision making authority available to the worker (Table 1).

**Table 1:** Basic components of R. Karasek JSQ model

**Component**	**Demand**
Decision latitude	
Skills discretion	Job requires learning new things
	Job requires high level of skills
	Job requires creativity
	Job entails a variety of things to do
	
Decision authority	Job allows making one's own decision
	Job provides a lot of freedom as to how the work gets done
	
Job demands	Job requires very hard work
	Job requires very fast work
	Job requires excessive work
	Job involves conflicting demands
	Jon involve not having enough time to get the job done

Skill discretion, measured by six items such as "keep learning new things', "can develop skills", "job requires skills", "task variety", "repetitious", and "job requires creativity", and decision authority, measured by three items such as "have freedom to make decisions", "choose how to perform work", and "have a lot of say on the job".

The second scale is psychological job demands, defined by five items such as "excessive work", "conflicting demands", "insufficient time to work", "work fast", and "work hard".

A four point Likert – like scale was used with the coding from 4 to 1 for series, so that the responses were summarized to give a score [46].

Data were also collected on supplementary aspects of stress and work characteristics, including: practice characteristics (partnership size, workplace location, patient load); and personal characteristics (gender, age, marital status).

### Statistical analysis

The data were computed – coded and analyzed using Statistical Package for the Social Sciences for Windows version 11.0 (SPSS Inc). The analysis included descriptive statistics, interrelation analysis and multivariate logistic regression as useful tool to predict the presence or absence of a characteristic or outcome based on values of a set of predictor variables. Logistic regression coefficients were used to estimate the odds ratios for each of the independent variables in the model.

Nonparametric tests were used to test for significant differences at the p = 0.05 level.

## Results

### Descriptive statistics

Of the 197 respondents, 162 (82.2%) GP's were female, and 35 (17.8%) male. The GP ages ranged from 31 to 66 years (mean 44.2 years, 95% CI 42.9 – 45.4). This reflects to the whole GP population in Lithuania. Significant gender difference was found for mean age (males 47.1 years, 95% CI 43.5 – 50.7; females 43.5 years, 95% CI 42.2 – 44.9; p < 0.03). All descriptive measures are shown in Table 2.

**Table 2:** Characteristics of general practitioners who responded to questionnaire survey of stress in general practice (N = 197).

**Variables**	**Number**	**%**
Gender		
Male	35	17.8
Female	162	82.2
Age (years)		
less 45	90	45.7
45–54	85	43.1
more 54	22	11.2
Years worked as GP		
less 8	40	20.3
8–28	115	58.4
more 28	42	21.3
Practice ownership type		
Solo practice	56	28.4
Group practice	141	71.6
Workplace		
City	123	62.4
Rural	74	37.6
Patient load (patient/day)		
less 18	21	10.7
18–28	140	71.1
more 28	36	18.3

### Prevalence of psychosocial stress among GP's by sociodemographic characteristics

Fourthy-eight percents of respondents could be classified as suffering from work related psychosocial stress by the Reeder scale. In figure 1 we see that there are considerable variations in psychosocial stress measures regarding to sociodemographic characteristics of GP's. The highest percentage of psychosocial stress by sociodemographic characteristics was found among widowed, single and female GP's.

### Work demands and decision latitude influence presence of psychosocial stress among GP's

**Figure 2:**
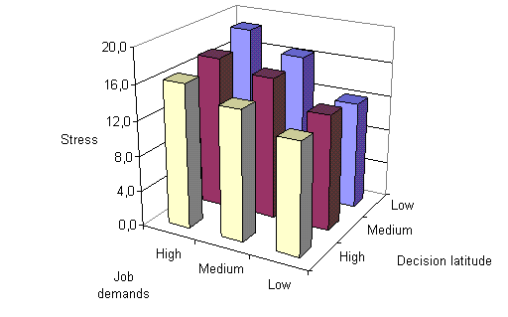
Interrelationship between job demands, decision latitude and psychosocial stress (χ^2 ^= 18,9; p < 0,01).

The job strain model suggests that high job demands, and low job control, are indicators of psychosocial stress. In figure 2 showed statistically significant interrelationship between job demands, decision latitude and psychosocial stress. Our results confirmed Job strain model's hypothesis that psychosocial stress occurs when job demands are high and job decision latitude is low.

### Predictors of psychosocial stress among GP's

The multivariate analysis (Table 3) shows that gender, work place location, practice ownership type, low ability to use skills and low decision latitude did not exhibit a statistically significant effect on psychosocial stress caseness and did not have a significant effect even when no other variables were controlled for. The model highlighted high job demands, patient load more than 18 patients per day and young age of GP's as significant predictors for psychosocial stress.

**Table 3:** Multivariate logistic regression model to predict psychosocial stress among Lithuanian GP's (n = 197)

Variables	B	P-value	OR	95,0% CI for OR
Constant	-4.782	<0,001		

Female gender (male – reference)	0,465	0,337	1,593	0,616–4,117
Rural workplace (city – reference)	0,261	0,478	1,298	0,632–2,665
Solo practice (Group practice – reference)	0,342	0,373	1,408	0,664–2,987
Age less 45(reference to age group 45–54)	1,928	0,024	6,874	1,292–36,582
Age more 54(reference to age group 45–54)	0,010	0,980	1,010	0,459–2,226
Practice duration 8–28 (more 28 – reference)	1,770	0,015	5,873	1,407–24,523
Practice duration less 8 (more 28 – reference)	1,627	0,054	5,089	0,975–26,552
Patient load 18–28 p/d (less 18 p/d -reference)	1,769	0,009	5,863	1,549–22,188
Patient load > 28 p/d (less 18 p/d -reference)	1,845	0,014	6,330	1,450–27,630
Low ability to use skills	0,198	0,609	1,219	0,571–2,600
Low decision latitude	0,317	0,343	1,373	0,713–2,644
High job demands	1,418	<0,001	4,128	2,102–8,104

OR – Odds Ratio

## Discussion

GPs are the professionals who are at the forefront of helping patients to manage urgent health problems, and as gatekeepers they have to make decisions on patient's health; whether to send them to hospitals. As a consequence of health care reform, GP's are required to have more competence in diagnosis and ongoing management of medical conditions. This means increased responsibilities, which may contribute to higher psychosocial stress for Lithuanian GP's. This can be regarded as stressors related to the new demands and sometimes it can interfere with personal life that can cause negative feelings about work, frustration, tension and lack of time to make appropriate decisions [47].

Our study has highlighted that fourthy-eight percents of respondents could be classified as suffering from work related psychosocial stress by the Reeder scale. Highest psychosocial stress prevalence among widowed, single and female GP's. Lowest psychosocial stress prevalence among was among males and older age GP's. The greatest risk to physical and mental health from stress occurs to GP's facing high psychological workload demands combined with low decision latitude in meeting those demands. High job demands, patient load more than 18 patients per day and young age of GP's can predict a statistically significant effect on psychosocial stress caseness. Gender, work place location, practice ownership type, low ability to use skills and low decision latitude does not exhibit for GP's in Lithuania a statistically significant effect on psychosocial stress development.

Main weaknesses of the present study can be mentioned: cross-sectional nature of the study, self reporting scales and generalisability of the questionnaire. Cross-sectional nature precludes an evaluation of temporal precedence and causality of the observed associations. Job Strain Model guided our hypotheses about causal relationships psychosocial stress and other work characteristics. The explored causal relations should be interpreted carefully and longitudinal studies should be carried out in the future research.

Second limitation – our reliance on self-reported rating scales can raise the issue of systematic positive or negative response tendencies. Several authors have argued that this phenomenon is not a major threat if interactions has been found [27,48]. Furthermore, as no scale is perfectly reliable, the associations between self-reported measures and self-reported workload appear to be weaker than they could be in reality.

Karasek's JCQ questionnaire was designed to be broadly applicable to a wide range of occupations, this means that factors that are specific to particular occupations may be overlooked. For example, job demands as it has been conceptualized and operationalised in this survey would not take into account some emotional demands that could be source of stress to GP's such as dealing with difficult patients or caring for the dying patients [30,31].

Otherwise, on the positive side, it is important to mention that generalisability of Karasek's model allows us to make comparisons among different medical and non-medical occupational groups and this has been an important factor in selecting the job strain model. Our results were obtained among a sample of people working in general practice. As strength of the investigation can be seen similar education level that respondents had. The sample size was sufficient regarding to sample size calculation and to allow exploration of tendencies. The participation rate was acceptable, and the scales used were previously validated instruments that retained their psychometric properties in our population. Findings from this research have hopefully emphasized the importance of examining changes and associations between work characteristics and psychosocial stress among GP's before health care reform in Lithuania will be definitely implemented.

## Conclusion

One half of respondents suffering from work related psychosocial stress. High psychological workload demands combined with low decision latitude has the greatest impact to stress caseness among GP's. High job demands, high patient load and young age of GP's can be assigned as significant predictors of psychosocial stress among GP's.

## Competing interests

The author(s) declare that they have no competing interests.

## Authors' contributions

GV designed the study, abstracted data, made data analysis, drafted and revised the manuscript.

SBA participated in initial study design, participated in data analysis and revised the manuscript.

All authors read and approved the final manuscript.

## Pre-publication history

The pre-publication history for this paper can be accessed here:


